# Hemagglutinin-neuraminidase and fusion proteins of virulent Newcastle disease virus cooperatively disturb fusion–fission homeostasis to enhance mitochondrial function by activating the unfolded protein response of endoplasmic reticulum and mitochondrial stress

**DOI:** 10.1186/s13567-019-0654-y

**Published:** 2019-05-22

**Authors:** Shanhui Ren, Zaib Ur Rehman, Mengyu Shi, Bin Yang, Panrao Liu, Yuncong Yin, Yurong Qu, Chunchun Meng, Zengqi Yang, Xiaolong Gao, Yingjie Sun, Chan Ding

**Affiliations:** 1 Department of Avian Infectious Diseases, Shanghai Veterinary Research Institute, Chinese Academy of Agricultural Science, Shanghai, 200241 China; 2 College of Veterinary Medicine, Xinjiang Agricultural University, Wulumuqi, 830052 Xinjiang China; 3 College of Veterinary Medicine, Yangzhou University, Yangzhou, 225009 Jiangsu China; 4 College of Veterinary Medicine, Northwest A&F University, Yangling, Shaanxi China; 5 College of Agriculture and Animal Husbandary, Qinghai University, Xining, 810016 Qinghai China; 6 Jiangsu Co-innovation Center for Prevention and Control of Important Animal Infectious Disease and Zoonoses, Yang Zhou, 225009 China

## Abstract

The fusogenically activated F and HN proteins of virulent NDV induce complete autophagic flux in DF-1 and A549 cells. However, the effect of both glycoproteins on mitochondria remains elusive. Here, we found that F and HN cooperation increases mitochondrial biogenesis but does not cause the mitochondria damage. We observed that both glycoproteins change the morphological characteristics and spatial distribution of intracellular mitochondria. F and HN cooperate cooperatively to induce ER stress and UPR^mt^. Our preliminary data suggested that F and HN cooperatively disturb mitochondrial fusion–fission homeostasis to enhance mitochondrial biogenesis, and eventually meet the energy demand of syncytium formation.

## Introduction, methods, and results

Autophagy is a conserved catabolic process that may be selective or nonselective. It delivers cytoplasmic contents, including damaged mitochondria and foreign pathogens engulfed into specific double-membrane autophagosome vesicles, to vacuoles or lysosomes for degradation and cycling [1]. The selective elimination of damaged mitochondria is termed as mitophagy, which is a type of macro-autophagy [1]. Mitophagy contributes to the maintenance of a healthy mitochondrial network pool and the prevention of programmed cell death. Some viral proteins, such as the tat of HIV [2], impair mitochondrial homeostasis to inhibit virus replication. Other viral proteins, such as the HBx of HBV [3], manipulate mitochondria to benefit viral propagation. However, the roles of the fusion (F) and hemagglutinin-neuraminidase (HN) glycoproteins of Newcastle disease virus (NDV) in mitochondrial have yet to be studied. Our previous study suggested that the F and HN of virulent NDV synergistically induced significant syncytium formation accompanied with complete autophagic flux in DF-1 and A549 cells [4]. As describe in our previous data [4], A549 cell line can be used as a cell model to study the function of both NDV glycoproteins (Flag-F and HA-HN) of NDV, which are constructed on the basis of the sequence of an F48E9 strain (Genbank Accession Number: MG456905), a standard velogenic strain. Considering the major roles of mitochondria in cellular functions, including suppling energy, regulating calcium levels, and controlling apoptotic cell death etc., we here investigated the relationship between the mitochondria and syncytium formation or membrane fusion induced by F and HN cooperation.

**Figure 1:**
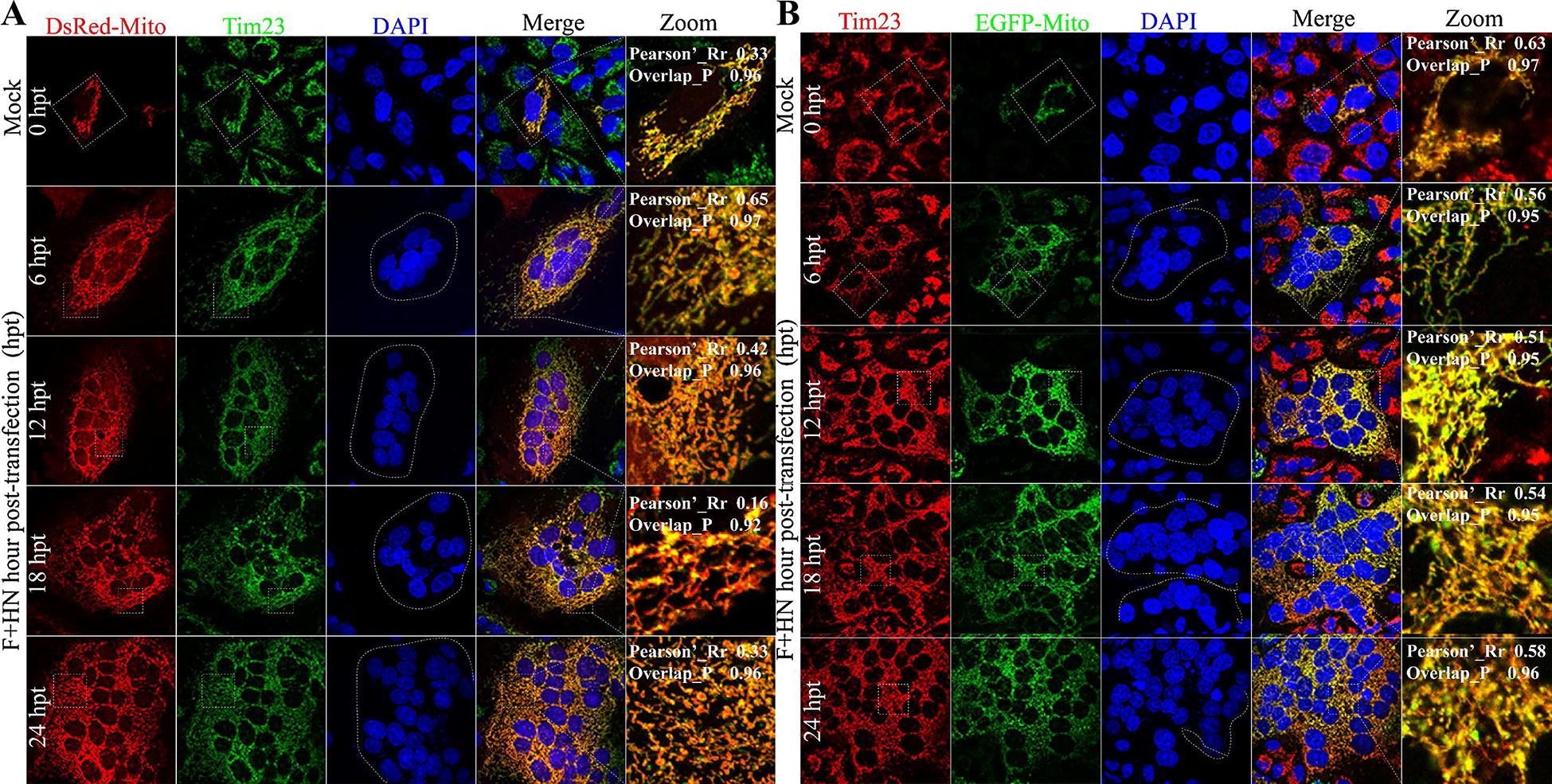
**F and HN co-expression does not cause mitochondrial damage rather than maintaining its integrity. A** Representative images of the colocalization between DsRed-Mito and endogenous TIM23 response to F and HN co-transfection. A549 cells were transfected with both viral plasmids (Flag-F and HA-HN) and DsRed-Mito. At 0, 6, 12, 18 and 24 hpt, the coverslips were examined via indirect immunofluorescence assay (IFA) in accordance with previously described protocol [4]. Syncytia formation can be observed on the basis of nuclei and indicated by a white dot circle. Blue (Nuclei), Green (endogenous TIM23), Red (mitochondria) and Yellow (Red + Green) denote overlapping of markers. The Person’s Rr, and the Overlap_R of zoomed out section were revealed, and both of them were calculated using Image J (National Institutes of Health, Bethesda, MD, USA). Pearson’s_Rr ranged from − 1.0 to 1.0, where 0 indicated no significant correlation and − 1.0 implied a complete negative correlation. Overlap_P ranged from 0 to 1.0, where 0.5 denoted that 50% of both selected channels were colocalized. **B** Representative images of colocalization between GFP-Mito and endogenous Tim23 response to F and HN co-transfection. A549 cells were transfected with both viral (Flag-F and HA-HN) and EGFP-Mito plasmids. Syncytium formation could be observed on the basis of nuclei and indicated by a white dot circle. All of the coverslip samples were collected at the corresponding marked time points, and this procedure was repeated at least twice. At 0, 6, 12, 18 and 24 hpt, the coverslips were examined via IFA in accordance with previously described protocol [4]. Blue (Nuclei), Green (mitochondria), Red (endogenous TIM23) and Yellow (Red + Green) denote overlapping of markers. The Person’s Rr, and the Overlap_R of zoomed out section were revealed, and both of them were calculated using Image J (National Institutes of Health, Bethesda, MD, USA).

**Figure 2:**
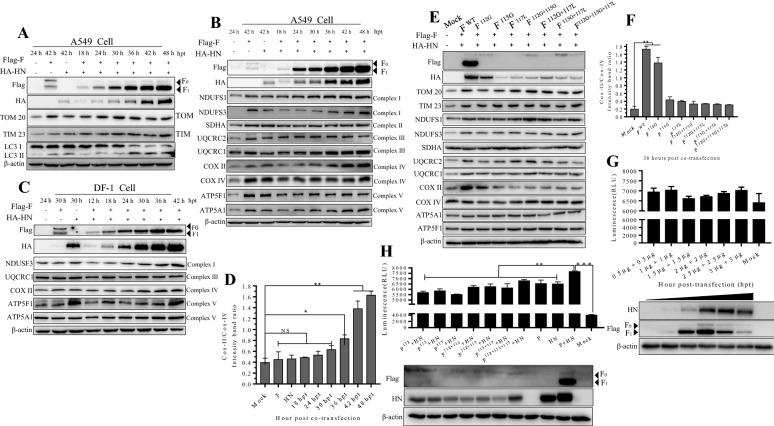
**F and HN cooperate synergistically to enhance mitochondrial biogenesis. A** Detection of TOM 20 and TIM 23 proteins through Western blot analysis in A549 cells. Western-blot samples were collected and performed in accordance with a previously described protocol [4]. **B** Detection of five membrane-bound protein complexes through western blot analysis in A549 cells. **C** Western blot analysis in DF-1 cells. **D** Intensity ratio of COX II to COX IV in response to F and HN transfection in A549 cells. The intensity of the COX II signal was normalized to the signal of COX IV subunit and calculated as the COXII: COIV ratio. Protein band intensities were quantified using Image J (National Institutes of Health, Bethesda, MD, USA). Significance was analyzed using a one-tailed Student’s *t* test. *p-value < 0.05; **p-value < 0.01. **E** Influence of the cleavage site motif of F on mitochondrial-related protein. In A549 cells, wild-type HN plasmid was co-transfected with wild-type F, F^112G^, F^115G^, F^117L^, F^112G+115G^, F^112G+117L^, F^115G+117L^ and F^112G+115G+117L^. At 36 hpt, the Western-blot samples were collected and analyzed. **F** Intensity ratio of COX II to COX IV in response to the co-transfection of eight F mutants and HN plasmids in A549 cells. Protein band intensities were quantified using Image J. **G** Synergistic cooperation of F and HN to enhance intracellular ATP levels. At 24 hpt, the intracellular ATP levels were determined using an enhanced ATP assay kit (Beyotime Biotechnology, S20027). The number of cells of the detected samples was normalized through Western blot analysis. **H** Influence of cleavage site motif on the intracellular ATP levels. In A549 cells, wild type HN plasmid was co-transfected with wild-type F, F^112G^, F^115G^, F^117L^, F^112G+115G^, F^112G+117L^, F^115G+117L^ and F^112G+115G+117L^. At 24 hpt, the ATP values of cells were determined using an enhanced ATP assay kit.

Most mitochondrial proteins are synthesized by a nuclear genome as precursors in the cytoplasm and imported across mitochondrial membranes by the translocase of protein complexes [5]. This translocase facilitates the importation of proteins through the translocation of the outer membrane (TOM) complex. The complementary translocase of the inner membrane (TIM) complex is responsible for protein transport across the inner membrane and into the mitochondrial matrix [5]. The TIM complex mainly includes the membranes of the mitochondrial carrier family of proteins. The TIM23 complex facilitates the translocation of matrix-targeted proteins in the mitochondrial matrix, where they must reside to function [6]. We initially hypothesized that the F and HN co-expression of virulent NDV may induce damage to mitochondria and further degrade them via selective mitophagy. To clarify whether intracellular mitochondria still maintain integrity with syncytium formation and membrane fusion, we first determined the distribution of intracellular TIM 23 response to both glycoproteins co-expression in A549 cells via indirect immunofluorescence assay (IFA). We then examined the colocalization between intracellular TIM 23 and mitochondria marked by two specific targeted technical plasmids, namely, DsRed-Mito (Figure 1A) and EGFP-Mito (Figure 1B). Unexpectedly, we found that their colocalization was accompanied with the extended co-transfection time of F and HN, suggesting that intracellular mitochondria had structural and functional integrity (Figure 1). Statistically, we calculated the Pearson’s correlation coefficient (Pearson’s_Rr) and the Overlap coefficient (Overlap_R) of the zoom out section by using the Image J to assess the correlation of the intensity distribution between channels and the true degree of co-location, respectively. Given the Pearson’s_Rr and Overlap_R (Figure 1), we confirmed that the co-expression of both glycoproteins did not cause intracellular mitochondria damage. To further determine whether selective mitophagy was induced by both glycoproteins, we performed Western blot assay to examine the intracellular protein level of TIM23 and TOM20, which is a TOM protein. In Figure 2A, both protein levels were slightly increased compared with those of mock control, suggesting that intracellular mitochondria were not damaged. Meanwhile, wildtype F and mutant F groups did not also impair the expression of both proteins at 36 h post-transfection (hpt), compared with those of control group. These results suggested that F and HN cooperation neither caused mitochondria damage nor induced mitophagy in A549 cells.

Mitochondria, the main energy hub of the cells, generate a constant flow of electrons through multiple parallel and alternative pathways that eventually end up in the mitochondrial electron transport chain. In mammals, the oxidative phosphorylation machinery occurs in the mitochondrial electron transport chain and is catalyzed by five membrane-bound protein complexes, namely, NADH-ubiquinol oxidoreductase (Complex I), succinate-ubiquinol oxidoreductase (Complex II), ubiquinol–cytochrome c oxidoreductase (Complex III), cytochrome *c* oxidase (COX) (Complex IV), and ATP synthase (Complex V) [7–9]. To further confirm the effect of both NDV glycoproteins on the mitochondria, we determined some subunits of the five protein complexes through Western blotting assay. As expected, all of the detected protein levels did not decrease in response to the F and HN co-expression in A549 cells (Figure 2B) and DF-1 cells (Figure 2C), suggesting that the mitochondria were not damaged, thereby maintaining their structural integrity. NDUSF1, NDUSF3, SDHA, UQCRC2, COX II, COX IV, ATP5A1 and ATP5F1 increased, especially NDUFS1, NDUFS3, COX II, COX IV, and ATP5A1, compared with those in the mock control, indicating that F and HN co-expression might influence the five membrane-bound protein complex functions. TOM20, NDUFS1, NDUFS3, UQCRC1, UQCRC2, COX II, COXIV, and ATP5F1, especially COX II (Figure 2E), in the wildtype F group increased at 36 hpt, compared with those in the control group and mutant group. Those data suggested that activated F_1_ and HN co-expression did not damage the integrity and function of intracellular mitochondria. Previous studies suggested that the ratio of the mitochondrial DNA (mtDNA) encoded subunit II of COX subunit II (COX II) to nuclear DNA(nDNA)-encoded COX I subunit IV (COX IV) is a surrogate of the translation efficiency of mitochondrial respiratory chain and mitochondrial protein synthesis [10–12]. Thus, we normalized and calculated the relative ratio of protein gray density (COX II to COX IV) by using Image J. In Figure 1D, we observed a time-dependent increase in the ratio of COX II to COX IV, suggesting the co-transfection of both glycoproteins enhanced the translation efficiency of mitochondrial respiratory chain and protein synthesis. In addition, the ratio of COX II/COX IV of the wildtype of F group was higher than that of mutant control group and mock group (Figure 2F), suggesting that the cleavage site motif of F influenced the translation efficiency of the mitochondrial electron transport chain. We determined the change in the intercellular adenosine 5’-triphosphate (ATP) level change via luciferin-luciferase bioluminescence method by using an enhanced ATP kit (Beyotime company, S0027). In Figures 2G and 2H, F and HN co-expression increase the intracellular ATP level. In short, F with HN cooperated synergistically to enhance mitochondrial biogenesis, including ATP production.

**Figure 3:**
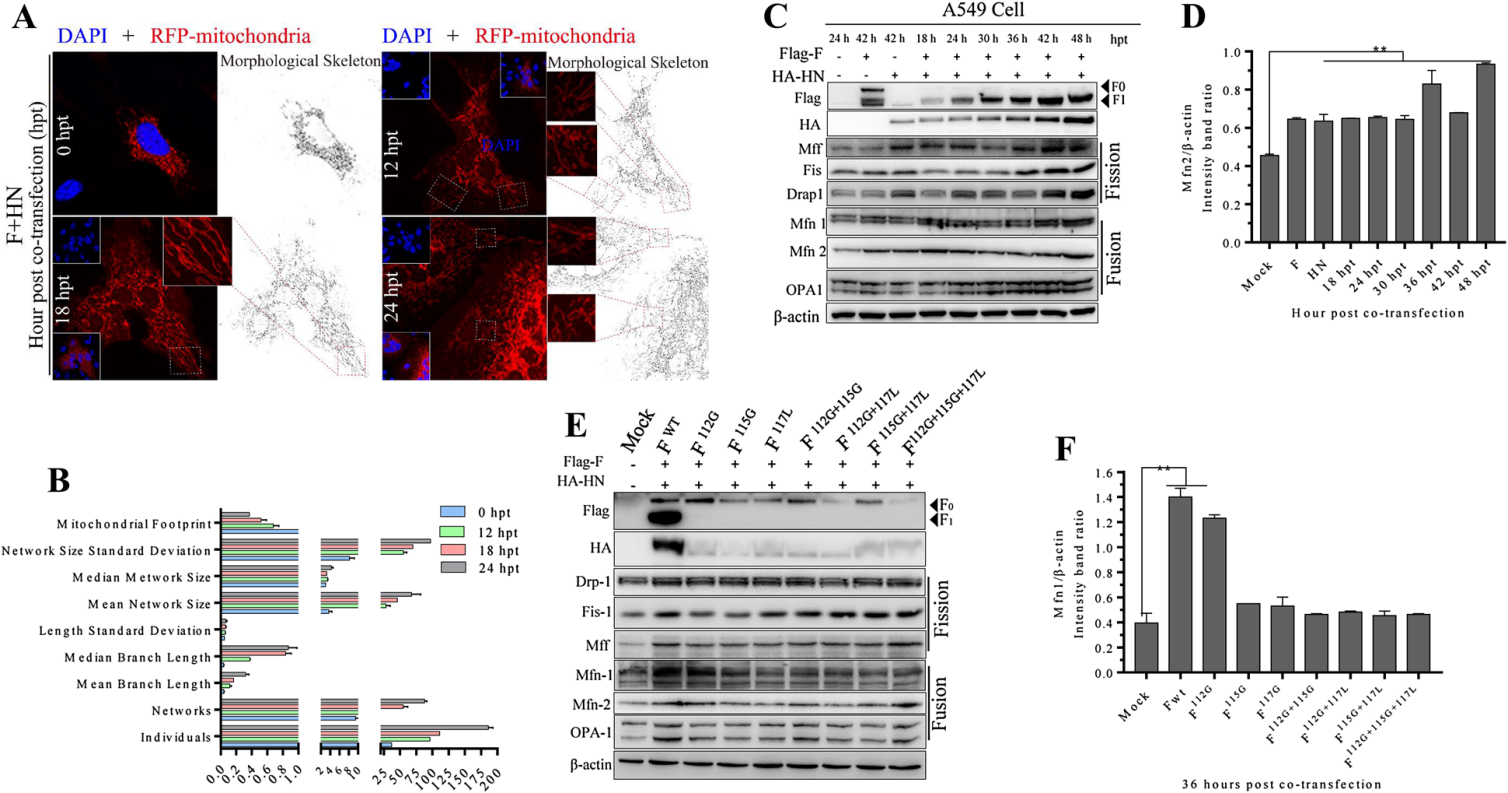
**F and HN cooperate synergistically to disturb mitochondrial fusion–fission homeostasis and induce nuclear aggregation. A** Representative images of F and HN co-operation that changes the subcellular distribution of mitochondria in A549 cells, which were mock transfected or transfected with F, HN, or both at the indicated time. At 0, 12, 18 and 24 hpt, coverslips were examined via IFA in accordance with previously described protocol [4]. After transfection, the treated cells were fixed, and processed for IFA. Image J was used to analyze the mitochondrial network morphological characteristic. **B** Results from mitochondrial network analysis performed in A549 cells. The mitochondrial network morphology was analyzed by using Image J. The workflow for the pre-processing and analysis of the targeted images was established in accordance with previously Ref. [15]. **C** Detection of fusion–fission proteins through Western blot analysis in A549 cells. **D** Intensity ratio of mfn2 to β-actin in response to F and HN co-expression in A549 cells. Protein band intensities were quantified using Image J. Significance was analyzed using a one-tailed Student’s *t*-test. *p-value < 0.05; **p-value < 0.01. **E** Influence of the cleavage site motif of F on the activation of ER UPR and UPR^mt^. In A549 cells, wild-type HN plasmid was co-transfected with wild-type F, F^112G^, F^115G^, F^117L^, F^112G+115G^, F^112G+117L^, F^115G+117L^ and F^112G+115G+117L^. At 36 hpt, the Western-blot samples corresponding to the marked point times were collected and analyzed. **F** Intensity ratio of Mfn1 to β-actin in response to the co-transfection cleavage mutant of F and HN in A549 cells at 24 hpt. Protein band intensities were quantified using Image J.

**Figure 4:**
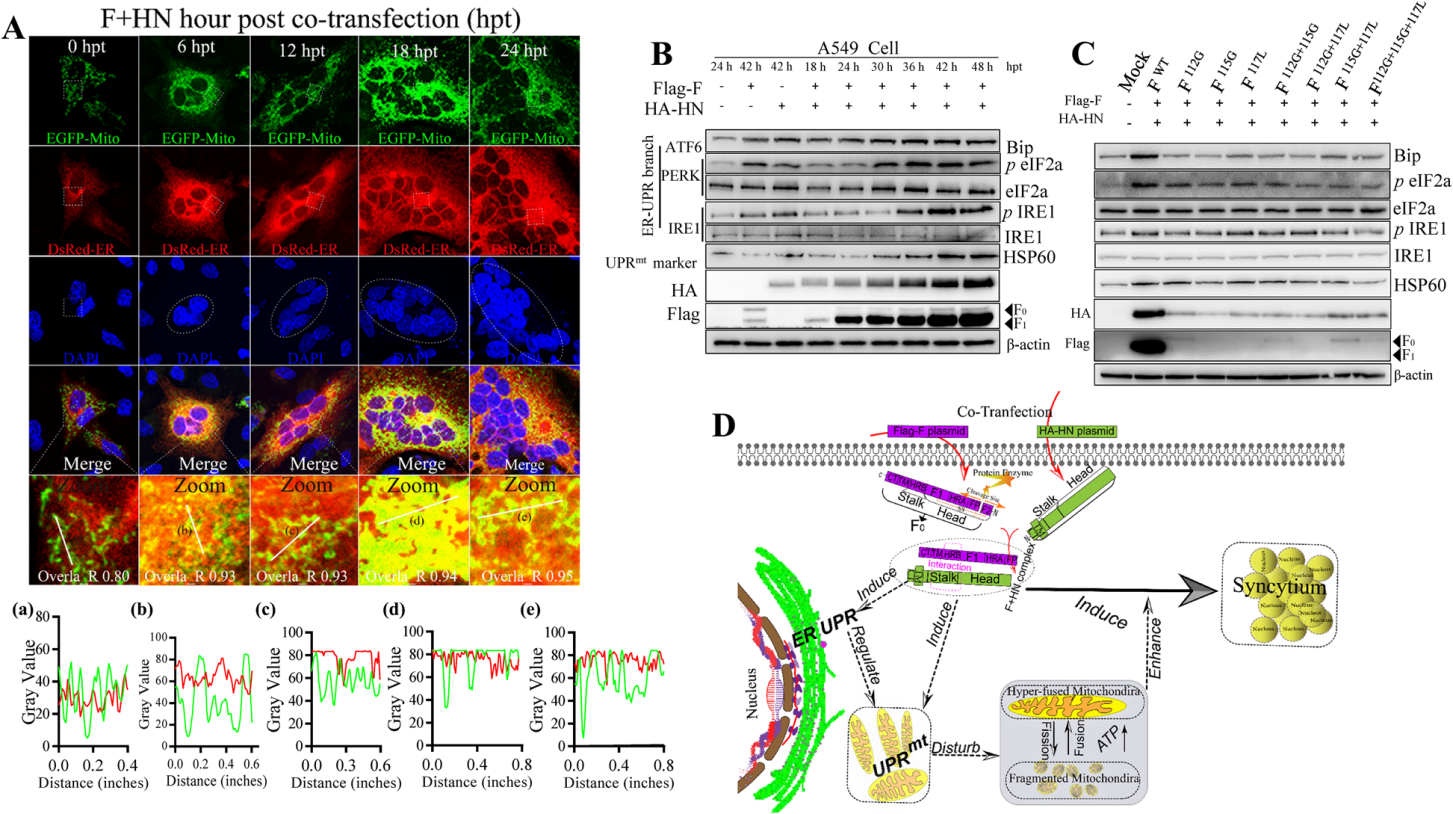
**Syncytia generated by HN and F of virulent NDV induce ER stress and UPR**^**mt**^. **A** Representative images of F and HN co-operation that increased the interaction between ER and mitochondria in A549 cells. A549 cell were co-transfected with EGFP-Mito and DsRed-ER, together with both Flag-F and HA-HN plasmids, at the indicated time. At 0, 6, 12, 18 and 24 hpt, the coverslips were examined IFA. The plot profile of linear region of interest was analyzed using Image J. **B** Detection results of ER stress and UPR^mt^ marker proteins in A549 cells through Western blot analysis. **C** Influence of the cleavage site motif of F on the activation of ER UPR and UPR^mt^. In A549 cells, wild-type HN plasmid was co-transfected with wild-type F, F^112G^, F^115G^, F^117L^, F^112G+115G^, F^112G+117L^, F^115G+117L^ and F^112G+115G+117L^. At 36 hpt, the Western-blot samples were collected and analyzed. **D** A model pattern of activated F_1_ interacts with HN for the disturbance of mitochondrial fusion–fission homeostasis to enhance syncytium formation via the UPR of ER and UPR^mt^. After HN and F co-transfection, the non-fusogenically F protein precursor (F_0_) form is proteolytically cleaved into a disulfide-linked F_1_ + F_2_ heterodimer, to be fusogenically active, which is an essential for fusion by positioning the hydrophobic fusion peptide (FP) at the newly formed N-terminus of F_1_ [29, 30]. Then, the fusogenically active F protein and HN form an F + HN complex via the interaction between a stalk and a domain with two heptad repeat regions (HRA and HRB) [29, 30]. Once triggered, HRB completely refolds around HRA, thereby forming six stable HBs and a fusion pore [29, 30]. Apart from spatial distribution rearrangement, the UPR of ER and UPR^mt^ is induced by activated F1 and HN to regulate and disturb mitochondrial fusion–fission homeostasis, which eventually produces hyper-fused and fragmented mitochondria. Eventually, nuclear-aggregated and hyper-fused mitochondria provide additional energy to enhance syncytia formation and membrane fusion.

The morphological characteristics and spatial organization of mitochondria are keys to mitochondrial quality control at protein and sub-organelle levels [13]. As shown in Figure 1A (vertical lines 1 and 5) and Figure 1B (vertical lines 7 and 10), F and HN synergistically altered cellular mitochondrial distribution in a time-dependent manner. To further identify the morphological characteristics and spatial distribution of cellular mitochondria, we conducted a time-course experiment to analyze mitochondrial movement by using DsRed-Mito plasmid (Figure 2A). We examined the mitochondrial network by obtaining original fluorescence microscopic images. The nuclear aggregation of the mitochondria was observed (Figure 3A), and this finding was consistent with the results shown in Figures 1 and 4A, suggesting that the functional interaction of both glycoproteins changed the subcellular movements and spatial distribution of intracellular mitochondria. Normal mitochondria are dynamic organelles, and them form interconnected tubular networks [13, 14]. The alteration of mitochondrial distribution is often accompanied with morphological and functional changes. Considering the aggregated mitochondria around syncytium formation site (Figure 1 vertical lines 1 and 5, Figures 3A, 4A horizontal line 1), we inferred that the spatial distribution of the mitochondria facilitated syncytium formation and membrane fusion induced by both glycoproteins. To obtain more accurate and specific results, we objectively analyzed the mitochondrial network morphology by using Image J as mentioned by Valente et al. [15]. On the basis of the fluorescent microscopy (Figure 3A) and the morphological skeleton analyses of the mitochondria (Figure 3B), we found that F and HN co-expression induced the fragmentation and hyper-fusion of the mitochondria in time-dependent manner. This observation was consistent with fluorescence results of Figure 1 (vertical line 1 and line 7) and Figure 4A (horizontal line 1). In general, mitochondrial elongation process is associated with the dimerization and activation of the ATPase function to produce additional energy [13, 16]. Considering the positive relationship of syncytium formation and the hyper-fusion of mitochondria in a time-dependent manner (Figure 1 vertical lines 1 and 7, Figures 3A, B, 4A horizonal line 1), we speculated that F and HN co-operation induced hyper-fusion of the mitochondria to maintain the energy demand of syncytium formation and membrane fusion. Mitochondrial homeostasis, including its architecture and functions, is maintained by two interlinked but distinct processes, namely, fission and fusion [17]. Mitochondrial fusion involves two sets of key GTPase proteins in mammals: the TOM mitofusin (Mfn) GTPases (Mfn1 and Mfn2) [18] and the TIM membrane optic atrophy 1(OPA1) [19], which is a dynamin-related guanosine triphosphatase mutated in dominant optic atrophy. The Mfn GTPases (Mfn1 and Mfn2) mediate TOM fusion, whereas the OPA1 mediates TIM fusion and crista integrity, resulting in the concomitant mixing of the mitochondrial contents and the merging of two individual mitochondria. To further assess whether F and HN change fusion–fission processes, we examined the related fusion protein changes through Western blot. The co-expression of both glycoproteins could increase Mfn1, Mfn2, and OPA1 protein levels (Figures 3C and D), especially Mfn1 and OPA1, compared with those of the control group, suggesting that mitochondrial fusion activity was stimulated. We also observed that the cleavage activity of F influenced the TIM and TOM fusion processes (Figures 3E and F). Mitochondrial fission is a complex process, including two distinct steps, namely, initial mitochondrial membranes constriction and membrane scission. Mitochondria membrane scission is mainly regulated by cytosolic dynamin-related protein 1 (DRP1), a member of the dynamin superfamily of GTPases [20]. The recruitment of DRP1 from the cytoplasm to the mitochondrial surface is a key step in fission regulation in mammalian and yeast cells. Once recruited to the mitochondria, DRP1 oligomerizes as rings on mitochondrial tubules and causes the scission of TOM and TIM. The recruitment of DRP1 in mammalian cells requires several accessory proteins, such as the mitochondrial fission protein 1 (Fis-1) and mitochondrial fission factor (Mff) [21]. Here, we also examined the changes in the levels of related fission proteins by using Western blot technique. F and HN proteins significantly increased Mff, Fis1, and Drap1 levels compared with those of the control group (Figure 3C), suggesting that the co-expression of both glycoproteins could increase the mitochondrial fission activity. Those data indicated that F and HN synergistically disturbed intracellular mitochondrial fusion–fission homeostasis, thereby stabilizing of membrane potential and mixing of matrix proteins, and this observation was consistent with the findings in Figure 1 (vertical line 1 and line 7). The cleavage site of the mutant of F influenced the intracellular fusion–fission protein level (Figure 3E). Therefore, the co-expression of both glycoproteins of NDV synergistically altered the fusion–fission homeostasis of intracellular mitochondria. However, the complicated regulatory mechanism should be further elucidated in future research.

Endoplasmic reticulum (ER) and mitochondria are dynamic, and they undergo continuous structural and spatial reorganization in response to cellular stress [22]. The ER-mitochondria contract serves as a platform for inter-organellar communication, which is necessary to regulate mitochondrial bioenergetic and function [22]. In response to various stresses, cellular ER physiology is mainly regulated by unfolded protein response (UPR), which consists of three integrated signaling branches that activate the downstream of the transmembrane ER stress sensor proteins: inositol requiring enzyme 1 (IRE1), activating transcription factor6 (ATF6), and double-stranded RNA-activated protein kinase(PKR)-like ER kinase (PERK) [23, 24]. Each pathway uses a different mechanism of signal transduction to drive the transcription of UPR target genes, such as ATF6 branch by regulated proteolysis, PERK branch by translational control, and IRE1 branch by nonconventional mRNA splicing [24]. The molecular quality control of mitochondria is regulated through mitochondrial UPR (UPR^mt^) response to various stresses, an adaptive response to proteotoxic stress, specifically in mitochondria [25, 26]. HSP60 is a mitochondrial chaperone responsible for the transportation and refolding of proteins from the cytoplasm to the mitochondrial matrix [27]. HSP60 is also generally used to evaluate the activation state of the UPR^mt^ [28]. Here, we speculated that activated F1 and HN induced ER stress and UPR^mt^ accompanied with membrane fusion and syncytium formation. To test this hypothesis, we co-transfected EGFP-Mito, DsRed-ER (a specific targeted ER plasmid), and both glycoproteins into A549 cells. In Figure 4A (horizontal line 4), excessive F and HN co-expression increased the interaction and tether between ER and mitochondria (yellow denoted colocalization), indicating the re-organization movement and spatial distribution of both organelles. The values of Overlap_R and plot profile also suggested that F and HN co-expression increased the true degree of co-location between the ER and the mitochondria. Western blot analysis revealed that activated F1 and HN synergistically stimulated the phosphorylation of IRE1 (IRE1 signal pathway transducer), eukaryotic initiation factor 2α (eIF2α, a PERK signal pathway transducer), and Bip (also known as GRP78, a chaperone of the heat shock protein [HSP]70 family) (both belong to ATF6 signal pathway transducers) [24, 26] (Figure 4B). This finding suggested that the overexpression of both glycoproteins could cause ER stress by activating the three branches of the UPR signaling pathway. On the contrary, cleavage site mutation influenced ER stress activation, indicating that the membrane fusion and syncytium formation ability determined the ER stress activation (Figure 4C). To assess UPR^mt^, we determined the protein change of HSP60 in response to the co-transfection of both glycoproteins through Western blot analysis. The protein expression of HSP60 was significantly increased in a time-dependent manner (Figure 4B), indicating UPR^mt^ activation. Similarly, we found that the cleavage ability of F influenced the UPR^mt^ activation (Figure 4C). On the basis of our results, we proposed a working model of the regulatory mechanism of mitochondrial homeostasis by the HN and F of NDV. Synergistic disruption of fusion–fission homeostasis by HN and F of NDV enhance mitochondiral function by activating of ER UPR and UPR^mt^ (Figure 4D).

## Discussion

Mitochondria are dynamic organelles that maintain the fusion–fission homeostasis in response to intracellular and extracellular stresses [14]. The maintenance of mitochondrial fusion–fission homeostasis is a complex and multistep cellular process, which must be maintained and regulated at protein and sub-organelle levels. This study aimed to investigate the relationship between syncytia generated by of F and HN of virulent NDV and intercellular mitochondria. We found that both glycoproteins did not induce selective autophagy to degrade the damaged mitochondria. Instead, they increased the biogenesis of mitochondria, along with syncytium formation and membrane fusion (Figures 1, 3). On the basis of the positive correlation of microscopic and Western blot analyses (Figures 1, 3, 4A), we speculated that the co-expression of both glycoproteins changed the mitochondrial distribution and morphological characteristics by disturbing mitochondrial fusion–fission homeostasis to meet the interaction energy demand of syncytium formation and membrane fusion. In this process, host cells must precisely regulate the mitochondrial distribution and morphological characteristics to meet energy demand and maintain the interactive activities of F and HN cooperation. However, the elaborate molecular mechanism should be further investigated.

The ER stress can be indirectly transmitted to the mitochondria by altering the transfer of metabolites, such as Ca^2+^, or directly influencing the mitochondria via the PERK-mediated UPR [22]. In this study, we observed that F and HN co-expression could increase eIF2α levels in A549 (Figure 4B) and DF-1 cells (data not shown) in a time-dependent manner. This finding suggested that PERK-mediated UPR phosphorylated eIF2α subunit, leading to transient translational attenuation. Therefore, we speculated that the PERK-mediated ER stress integrated transcriptional and translational signaling to protect mitochondrial function, and this finding was consistent with the PERK-dependent remodeling of mitochondrial quality control pathways to prevent the potentially pathologic accumulation of misfolded or damaged proteins during ER stress [16, 22]. Recently, Lebeau et al. [16] reported that stress-induced mitochondrial hyper-fusion is triggered by PERK-dependent translation attenuation via the promotion of the electron transport chain, suggesting that cellular mitochondria could autonomously integrate by remodeling their morphological characteristics and distribution response to ER stress. We inferred that hyper-fusion was induced by co-expression of both glycoproteins via the PERK-mediated stress signaling pathway to maintain energy demand. However, the underlying mechanism should be elucidated in detail.

In summary, we at first reported that both glycoproteins of NDV neither damaged mitochondria nor induced mitophagy. The co-transfection of both viral glycoproteins changed mitochondrial spatial distribution and disturbed the mitochondrial fusion–fission homeostasis, along with syncytium formation and membrane fusion. To meet high-demand energy of syncytia formation, activated virulent F1 and HN synergistically enhanced mitochondrial biological function and eventually supplied energy by activating of UPR of ER and UPR^mt^. We speculated that the activated UPR of ER and UPR^mt^ might represent a cellular protective mechanism to facilitate the refolding of imported proteins and the proper assembly of unfolded polypeptides in the mitochondria and the ER. Overall, our preliminary data could help to elucidate the molecular mechanism of membrane fusion and syncytium formation induced by F and HN of NDV.
